# Sustainable Synthesis of Metal-Doped Lignin-Derived Electrospun Carbon Fibers for the Development of ORR Electrocatalysts

**DOI:** 10.3390/nano13222921

**Published:** 2023-11-09

**Authors:** Cristian Daniel Jaimes-Paez, Francisco José García-Mateos, Ramiro Ruiz-Rosas, José Rodríguez-Mirasol, Tomás Cordero, Emilia Morallón, Diego Cazorla-Amorós

**Affiliations:** 1 Departamento de Química Física, Instituto Universitario de Materiales de Alicante (IUMA), University of Alicante, Ap. 99, 03080 Alicante, Spain; cristian.jaimes11@ua.es (C.D.J.-P.); morallon@ua.es (E.M.); 2 Departamento de Ingeniería Química, Andalucía Tech, University of Malaga, Campus de Teatinos s/n, 29010 Malaga, Spain; garciamateos@uma.es (F.J.G.-M.); mirasol@uma.es (J.R.-M.); cordero@uma.es (T.C.); 3 Departamento de Química Inorgánica, Instituto Universitario de Materiales de Alicante (IUMA), University of Alicante, Ap. 99, 03080 Alicante, Spain

**Keywords:** electrospinning, carbon fibers, lignin, self-standing electrodes, ORR

## Abstract

The aim of this work is to establish the Oxygen Reduction Reaction (ORR) activity of self-standing electrospun carbon fiber catalysts obtained from different metallic salt/lignin solutions. Through a single-step electrospinning technique, freestanding carbon fiber (CF) electrodes embedded with various metal nanoparticles (Co, Fe, Pt, and Pd), with 8–16 wt% loadings, were prepared using organosolv lignin as the initial material. These fibers were formed from a solution of lignin and ethanol, into which the metallic salt precursors were introduced, without additives or the use of toxic reagents. The resulting non-woven cloths were thermostabilized in air and then carbonized at 900 °C. The presence of metals led to varying degrees of porosity development during carbonization, improving the accessibility of the electrolyte to active sites. The obtained Pt and Pd metal-loaded carbon fibers showed high nanoparticle dispersion. The performance of the electrocatalyst for the oxygen reduction reaction was assessed in alkaline and acidic electrolytes and compared to establish which metals were the most suitable for producing carbon fibers with the highest electrocatalytic activity. In accordance with their superior dispersion and balanced pore size distribution, the carbon fibers loaded with 8 wt% palladium showed the best ORR activity, with onset potentials of 0.97 and 0.95 V in alkaline and acid media, respectively. In addition, this electrocatalyst exhibits good stability and selectivity for the four-electron energy pathway while using lower metal loadings compared to commercial catalysts.

## 1. Introduction

One of the biggest challenges today is obtaining energy from renewable resources due to the rapid worldwide growth in pollution, greenhouse gas emissions, and dependence on fossil sources for energy. This demand has led to the development of new and promising electrochemical energy storage and generation technologies, such as fuel cells and redox flow or metal–air batteries, where the use of carbon nanomaterials as electrodes plays a crucial role [1,2,3]. Most of these devices share the oxygen reduction reaction (ORR) at the cathode as a common reaction. The sluggish kinetics of the ORR pose a significant challenge for the commercialization of fuel cells and metal–air batteries [4,5]. In addition, the current ORR electrocatalysts contain a high amount of Pt, whose scarcity and high cost hamper the implementation of this technology. Therefore, the development of sustainable, carbon-based electrocatalysts for the ORR with either a lower quantity of noble metals due to improved efficiency [6] or based on non-precious metals [7], and even in metal free, heteroatom-doped carbons [8,9], is of major importance and constitutes a central concern for the deployment of alternative energy sources.

Carbon materials can be obtained from renewable feedstocks, increasing the sustainability of the electrochemical devices in which they are applied. In this sense, lignin, one of the main components of the cell walls of plants, is a three-dimensional polymer consisting of interconnected phenylpropane units bonded together and is recognized as the richest source of renewable aromatic compounds across the globe [10]. Considering its low cost, high availability, and great potential, along with its elevated carbon content, lignin has sparked attention in relation to exploiting its use as feedstock for the preparation of carbon materials as adsorbents [11], carbon-based catalysts [12,13], and composite materials [14,15] and also for the preparation of conductivity promoters, supercapacitors, and Li-ion or even redox flow battery electrodes [16,17,18].

Lignin is usually obtained in the pulp and paper industry as a co-product of the cellulose isolation processes, in which it is burned to close the energy balance of a plant. In addition, lignin is also obtained as co-product in lignocellulose-based biorefineries where greener cellulose isolation methods, such as the Organosolv process, are introduced. In contrast to the most common Kraft lignin, Organosolv lignin contains minimum quantities of inorganic elements. It is obtained from lignocellulosic biomass through an extraction process carried out in non-toxic organic solvents, rendering the obtained lignin soluble in organic chemicals as ethanol or acetic acid. It also results in higher pulp yields and delivers a lignin product with a lower content of impurities through more environmentally friendly processes [19].

In the last few years, the production of controllable fibers has led to an increased interest in relevant fields such as renewable energy, especially in relation to storage devices and electrochemical energy conversion [20,21,22]. In this context, electrospinning is a versatile approach to preparing lignin-based nanofibrous materials, applying high voltage and selected polymer solutions of suitable viscosity and conductivity. It is ideal for preparing polymeric continuous fibers with diameters ranging between a few microns and a few tens of nanometers [10,20]. Most lignin-containing solutions exhibit low spinning capacity, making it necessary to add a plasticizing agent to prevent beading and facilitate electrospinning [23,24]. However, concentrated organosolv lignin solutions in ethanol are readily spinnable, as it is possible to obtain metal-doped lignin derivatives simply by adding the appropriate metal precursor into the lignin solution, which allows for better control of the final composition and properties of the fibers [25]. Such characteristics offer an optimal method for producing electrodes for a wide diversity of electrochemical devices, reducing mass transfer and overpotentials for activation and thus enhancing efficiency. In this context, electrospun carbon fibers have gained lots of attention for the development of ORR electrocatalysts due to their remarkable combination of enhanced mass transfer, electrical conductivity, chemical and mechanical stability, and easily controllable composition and structure [20,26]. However, most of the ORR electrospun carbon-based electrocatalysts currently reported have been prepared from non-renewable polymers, such as polyacrylonitrile. Replacing them with lignin would improve the sustainability of these electrocatalytic systems [20]. The preparation of ORR-oriented lignin electrocatalysts via electrospinning has been studied in previous works [27,28,29,30]. Nevertheless, the corresponding authors employed plasticizing additives for electrospinning; in some cases, costly activation methods were used, and the performance with respect to the ORR did not come close to that exhibited by commercial materials. 

In this study, a sustainable and simplified experimental method for obtaining electrospun lignin-based carbon fiber electrocatalysts via the electrospinning of lignin solutions with different metal salts in one step and without the use of any additives, followed by the stabilization and carbonization of the resulting non-woven mats, is proposed. The lignin-derived, metal-loaded electrocatalysts have relatively low proportions of Fe, Co, Pt, and Pd nanoparticles, which are very well distributed, and were used for the first time as electrocatalysts for the ORR. A detailed study of the physicochemical properties of the electrocatalysts was conducted to determine which one was the most suitable for the ORR and their possible future applications in devices such as fuel cells and metal–air batteries.

## 2. Materials and Methods

### 2.1. Electrocatalyst Preparation

**Figure 1:**
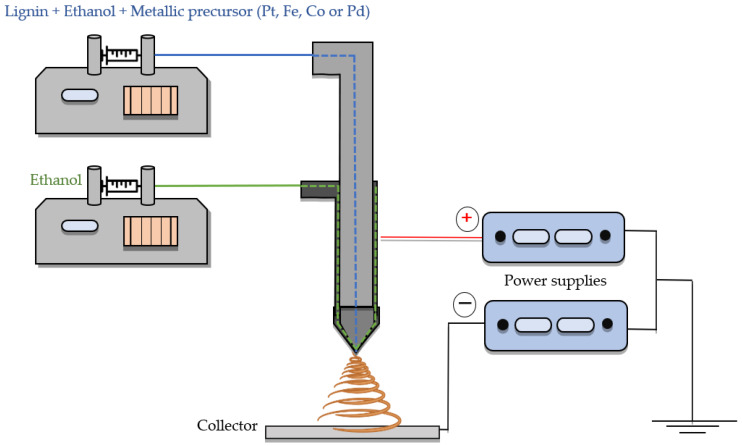
Scheme of the assembly used in the coaxial electrospinning configuration.

Lignin/ethanol solutions were prepared by mixing organosolv lignin (Alcell^®^) (Repap Technologies Inc., Vancouver, BC, Canada) and ethanol in a 1:1 mass ratio. Different metal precursors were added to this solution, allowing for the preparation of metal-doped-lignin fibers via electrospinning in just one step. For this purpose, platinum acetylacetonate (Pt(C_5_H_7_O_2_)_2_), palladium chloride (PdCl_2_), cobalt nitrate (CoNO_3_), and iron nitrate (FeNO_3_) (Sigma Aldrich, Darmstadt, Germany) were used as metal precursors, maintaining in all cases a metal (Pt, Pd, Fe, or Co)/lignin weight ratio of 0.015. Prior to the electrospinning process, the solutions were stirred at 250 rpm at 60 °C overnight. The co-axial electrospinning system used for the preparation of lignin fibers is shown in Figure 1. The applied electrical potential difference, determined by using two high-voltage power supplies, for the lignin fibers varied from 0.56 to 0.72 kV m^−1^. Feed rates for the lignin solution flowing through the inner needle varied from 1 to 3 mL h^−1^, and ethanol (needed to prevent Taylor cone solidification [31]) was pumped in using the outer needle, with a flow rate of 0.10–0.15 mL h^−1^. The flows were tuned to achieve continuous spinning and stable Taylor cone formation due to the fact that the addition of different metal precursors altered the conductivity, viscosity, and surface tension of the lignin solution. An oxidative thermostabilization step applied to the lignin fibers was carried out from 60 °C to 200 °C using a heating ramp of 5 °C h^−1^ in air atmosphere. The final stabilization temperature was maintained for 20 h. Carbonization was carried out at 900 °C under N_2_ flow and with a heating rate of 10 °C min^−1^. The carbon fiber electrocatalysts were named CF followed by the corresponding metal element.

### 2.2. Physicochemical Characterization Techniques

To determine the porous texture of the samples, nitrogen adsorption isotherms at −196 °C were developed (ASAP2020 apparatus of Micromeritics). The samples were degassed for 8 h (150 °C) under vacuum. The BET equation was used to determine the apparent surface area (A_BET_) from the nitrogen isotherms, and the t-method was used to calculate the volume of micropores (V_t_). The mesopore volume (V_mes_) was obtained via the subtraction of the micropore volume by the total pore volume at a relative pressure of 0.96 [32]. Using the SAIEUS program, 2D-NLDFT heterogeneous surface model was applied to the N_2_ adsorption isotherms, providing the corresponding micropore size distributions [33].

X-ray photoelectron spectroscopy (XPS) carried out using a Physical Electronics model 5700 C equipped with a magnesium anode as an X-ray source was employed to study the surface chemistry of the carbon fibers and the metal loadings. The peaks were corrected using the C1s peak at 284.5 eV, with the uncertainty of the equipment being ±0.2 atomic %.

Thermogravimetric system (CI Electronics) was employed to determine the inorganic matter content of the carbon fibers. In these experiments, 10 mg of carbon fibers was calcined at 900 °C using a heating rate of 10 °C min^−1^. The metal loading was determined from the resulting ashes, considering that metals were in the form of oxides. The composition of the ashes was analyzed using XRF in order to check the final loading on the carbon fibers.

Raman spectra data were acquired at 532 nm using a Jasco NRS-5100 spectrometer. X-ray diffraction (XRD) patterns of the electrocatalysts were recorded using a Bruker D8 Advance diffractometer (Billerica, MA, USA). The XRD patterns were measured using Cu Kα radiation source. Scherrer’s equation was used to determine the size of the crystallite according to the method used by Waseda et al. [34].

Finally, the surface morphology and structure of the metal-loaded carbon fibers were determined using transmission electron microscopy (TEM) (using FEI Talos F200X High-Resolution Transmission Electron Microscope) with a 200 kV FEG beam and scanning electron microscopy (SEM) (JSM-6490LV JEOL microscope) at a voltage of 25 kV. Using ImageJ software (V 1.53K), ~100 nanoparticles were measured in representative micrographs of each catalyst to determine the average nanoparticle size and particle size distribution. For the determination of the average diameter size of carbon fibers, SEM images were used. Specifically, for each type of fiber, five images were captured, and within each image, the average diameter was calculated based on measurements from 30 individual fibers.

### 2.3. Electrochemical Characterization

An Autolab PGSTAT302 potentiostat (Metrohm) was used for all the electrochemical measurements. Details about the configuration of the cell, reference electrodes, and rotating ring disk electrode can be found in the study by Flores-Lasluisa et al. [35].

The electrochemical responses of the electrospun carbon fiber materials in oxygen-free 0.1 M KOH and 0.5 M H_2_SO_4_ electrolytes were established using cyclic voltammetry (CV) applied in the range between 0 and 1.4 V (vs. reversible hydrogen electrode (RHE)). Several cycles were carried out before the measurement to activate and clean the catalytic sites. To perform the ORR study, the carbon fibers were cut to the same diameter as the glassy carbon disk of the rotating disk electrode and directly attached to the glassy carbon surface with a Nafion^®^ (Sigma-Aldrich, Darmstadt, Germany) solution at 0.2 wt%. The ORR catalytic activity was measured using Linear Sweep Voltammetry (LSV) tests conducted at 5 mV s^−1^ from 1.0 to 0 V (vs. RHE) in O_2_-saturated acid and alkaline media at several rotation speeds (between 400 and 2025 rpm). Throughout all tests, a constant potential of 1.5 V was maintained for the Pt ring electrode. The electron transfer number, n, was determined from the current generated by the oxidation of hydrogen peroxide at the ring electrode [35]:

(1) $$n=\frac{4I_{D}}{I_{D}+I_{R}/0.37}$$

In the equation above, I_R_ and I_D_ are the currents, as absolute values, determined on the disk and ring electrodes, respectively, and 0.37 is the collection efficiency of the RRDE, which was experimentally determined. 

For better electrochemical characterization and better control of the amount of catalyst used, inks were obtained from the fibers (see Supporting Information). This permitted improved contact with the glassy carbon collector. In these experiments, the glassy carbon disk was modified with the samples using a 1 mg mL^−1^ dispersion of each electrocatalyst (20% isopropanol and 0.02% Nafion^®^. The amount of catalyst on the disk electrode was optimized to attain the highest limiting current density, with 120 μg being the optimum value. 

The Electrochemical Surface Area (ECSA) is estimated from the double-layer capacitance (*Cdl*). To calculate it, cyclic voltammetry (CV) curves obtained in a potential range of 0.6–0.7 V vs. (RHE) were used for Fe, Co, and Pt fibers at sweep rates of 10–100 mV s^−1^. For the Pd fiber, a potential range of 0.8–0.9 V vs. RHE was used.

The value of *Cdl* was determined in reference to half of the slope of the *Ja*–*Jc* vs. scan rate plot), where *Ja* and *Jc* represent the anodic and cathodic currents, respectively, at a potential of 0.65 V vs. RHE in the case of Fe, Co, and Pt fibers and at a potential of 0.85 V vs. RHE for Pd fibers.

TOF was calculated using the current at 0.4 V, and the amount of metal was determined from XPS, assuming that this corresponded to the accessible metal (see Table S1). 

The impedance tests were carried out at a potential of 0.2 V vs. RHE using N_2_-saturated 0.1 M KOH. The frequencies ranged from 6000 to 0.1 Hz.

A stability test was carried out by subjecting the samples to 500 cycles in a potential range between 0.5 and 1.0 V, at a sweep speed of 100 mV s^−1^, in an O_2_-saturated atmosphere.

## 3. Results and Discussion

### 3.1. Carbon Fiber Preparation Yields

Table 1 presents the preparation yields of the different carbon-fiber-based electrocatalysts. The overall preparation yields range from 8% (CF-Co and CF-Fe) up to 17% (CF-Pd), with all the carbonization yields being consistently lower than the yields from the air stabilization steps. Weight loss during stabilization is attributed to the evaporation of enclosed ethanol as well as elimination reactions between oxygen functional groups generated by the air treatment, the former of which takes place during the cross-linking of lignin polymeric chains [36]. The presence of metallic salts seems to promote oxidation reactions to some extent [25]. Additionally, the decomposition of the utilized salts may contribute to some weight loss, although with different contributions considering their different decomposition temperatures; for example, the decomposition of metal nitrates occurs at lower temperatures than those for acetylacetonates and chlorides [37,38,39]. 

During the carbonization process, volatile carbon molecules exit the solid, together with most of the oxygen existing as surface oxygen groups that were formed during the stabilization step, resulting in condensed polyaromatic carbon structures in the finished product [40]. The carbon fibers with the highest carbonization yield were the palladium-containing carbon fibers, achieving a carbonization yield of around 30%, whereas for those loaded with cobalt and iron, a lower carbonization yield was achieved. This could be related to the self-gasification of the latter lignin fibers with evolved water and CO_2_ during the carbonization process, a phenomenon already reported during the carbonization of cobalt-containing lignin fibers owing to the catalytic effect of cobalt oxides and/or metallic Co [25] and further reactions with the carbon material.

The bulk metal loadings for all the electrocatalysts were determined, and they are reported in Table 2. Despite using the same metal-to-lignin ratio in the starting solution, the different degrees of weight loss induced during carbonization in each active phase led to different metal loadings on the final sample, ranging from 8 wt% (CF-Pd) to 16 wt% (CF-Fe). Note that the achieved amounts for the resulting Pd and Pt electrocatalysts (8 and 10 wt%) are lower than those used in commercial ORR catalysts (20–50 wt%).

### 3.2. Physicochemical Characterization

**Figure 2:**
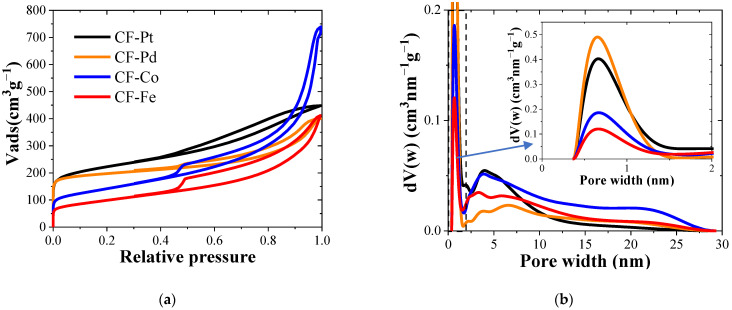
(**a**) N_2_ adsorption–desorption isotherms at −196 °C of carbon fibers. (**b**) Pore size distribution of the catalysts (inset: micropore distribution).

The N_2_ adsorption–desorption isotherms at −196 °C for various lignin carbon fiber electrocatalysts are shown in Figure 2a. All the samples exhibited type I-IV adsorption isotherms, with a hysteresis loop of types H3-H4 according to the IUPAC classification. In these isotherms, significant N_2_ uptake at low relative pressures was obtained, which is a feature characteristic of microporous solids. Moreover, all the samples presented increasing N_2_ adsorption at medium and high relative pressures, including a hysteresis loop during desorption, and these phenomena are consistent with the existence of mesopores, which could help to facilitate mass transport, thus boosting catalytic performance [41]. The samples exhibit well-developed BET surface area values between 351 and 786 m^2^ g^−1^, with the iron- and cobalt-containing carbon fibers having a lower value than the platinum- and palladium-containing carbon fibers. The generation of mesoporosity in the cobalt- and iron-containing fibers could be related to the combination of carbothermal reduction and phase transitions during carbonization. The formation of metal carbides has been reported to take place at pyrolysis temperatures between 400 and 600 °C in transition metal complexes such as iron and cobalt succinate and fumarates, releasing CO_2_ and H_2_O, while the metal carbides are reduced to a metallic phase at temperatures higher than 800 °C [42]. Differently, platinum and palladium do not experience such phase transition changes, generating only reduced metallic particles at high carbonization temperatures. For oxygen reduction applications, it is necessary to develop a large surface area that facilitates improved mass transfer and higher accessibility to the active centers, enhancing the electrocatalytic response. Then, it is expected that these materials may have interesting performance in this reaction since they combine both micrometer-size fibers and well-developed porosity.

The pore size distributions (PSDs) derived from the N_2_ adsorption isotherms are shown in Figure 2b. They confirm that all the samples present microporosity and mesoporosity, with a higher presence of micropores in the Pt- and Pd-containing carbon fibers. The Pd-containing sample attained the highest microporosity. This indicates that the carbon fiber samples prepared with Pd and Pt precursors allowed for some carbon gasification, thus enabling the development of a greater volume of micropores on the surfaces of the carbon fibers. The presence of mesoporosity is a desirable feature for electrocatalysts, improving the mass transfer rate of oxygen towards the active sites. The PSD distribution reveals the superior mesopore development in CF-Co for all the pore sizes, while CF-Fe shows a lower presence of wide mesopores (10–30 nm, Figure 2b). As for the mesopore sizes of the rest of the samples, CF-Pt presented a larger share of narrow mesopores (2–5 nm) than CF-Pd, which presented a broader distribution of sizes (5–30 nm) with a greater number of wide mesopores (10–30 nm) than CF-Pt.

Table 2 displays the porosity parameters for the prepared carbon fibers. The different salt precursors induced severe modifications of porosity development. Thus, during carbonization, the Pt and Pd electrocatalysts developed about equal quantities of micropores and mesopores. In contrast, using nitrate-based salts to prepare Co- and Fe-loaded fibers mostly led to the production of mesopores on the final carbon fibers. Previous works found that carbonized lignin fibers only develop narrow microporosity [43], pointing out the relevance of the addition of metal salts in the lignin solution in order to favor mesoporosity generation [10].

The mass surface concentrations of the different elements obtained via XPS are listed in Table 2. For comparative purposes, the metal loadings determined via XRF are also included. As can be observed, according to the data obtained via XPS for all the samples, the carbon amount was between 90 and 92%, with a low degree of oxygen (between 2 and 4%). On the other hand, the metal loading on the surfaces of the CF-Pd and CF-Pt samples was about 7%, which is almost twice that of the CF-Co and CF-Fe samples. Considering that Co- and Fe-containing fibers have a higher bulk metal loading (Table 2), it can be concluded that the Co and Fe particles were not fully exposed on the surface but remained in the inner regions of the fibers or covered by carbon layers, thus wasting the exposed surface, resulting in the absence of parts of their active sites for catalytic reactions. Contrarily, the same comparison of the XPS results with the bulk content for the Pt- and Pd-containing samples points to a more uniform distribution of the metals on the surface and in the bulk of the carbon fibers. In this case, the materials that present a closer surface-to bulk-concentration ratio are the Pd-containing carbon fibers.

**Figure 3:**
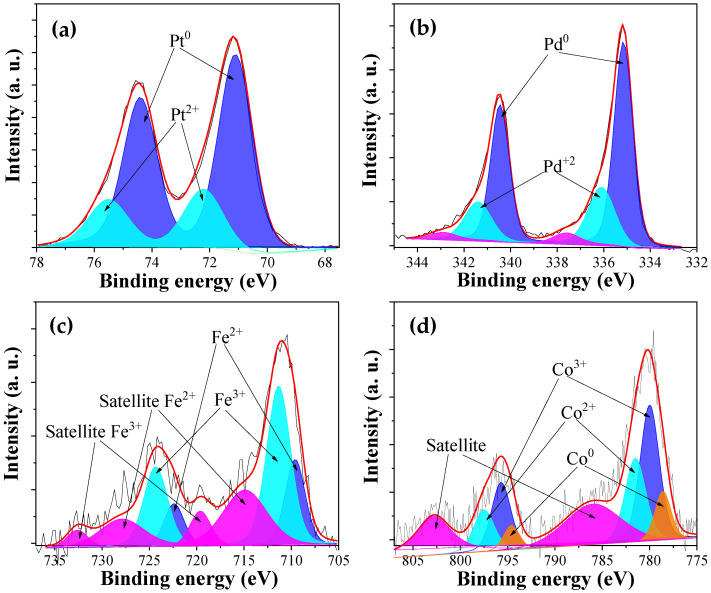
XPS spectra of the carbon fibers of different metallic precursors: (**a**) CF-Pt, (**b**) CF-Pd, (**c**) CF-Fe, and (**d**) CF-Co.

Figure 3 comprises the XPS spectra for each metal, which are representative of the carbon fibers studied. In the case of the noble metals (Figure 3a,b), it can be observed that the metallic state is predominant, while the (II) state was detected in a smaller proportion. This result confirms that the process of the synthesis of the carbon fibers results in a reduction in the number of metal nanoparticles. For platinum, Pt (0) was detected at binding energies of 71.1 and 74.4 eV for Pt 4f_7/2_ and 4f_5/2_, respectively [44], while, in the case of Pd, this metal species was detected at binding energies of 335.2 eV and 340.5 eV for 3d_5/2_ and 3d_3/2_, respectively [45]. Figure 3c shows the deconvolution of the XPS peaks for the case of the iron present in the iron-containing carbon fibers. The presence of metallic iron was not observed owing to the temperature reached in the synthesis of the carbon fibers being insufficient to reduce iron to its metallic form. In this case, for Fe 2p_3/2_, the presence of iron (II) and (III) at binding energies of 709.6 and 711.3 eV, respectively, can be observed, along with a doublet corresponding to spin–orbit coupling [46,47]. The characteristic satellites were also detected; i.e., a satellite peak centered at 715 eV is characteristic of Fe (II), while a satellite at 719.7 is characteristic of Fe (III). In the case of the cobalt-containing fibers, although Co (II) and Co (III) sites have similar 2p binding energies, when Co_3_O_4_ is formed, differentiation between the different states is possible [48]. A partial reduction in the synthesized nanoparticles and two shakeup satellites were observed, showing the coexistence of Co^2+^ and Co^3+^ in Co_3_O_4_. Figure 3d shows the deconvolution of the XPS spectrum for this metal into three main contributions for Co 2p_3/2_ at binding energies of 778.6 eV for metallic Co and at 780.0 eV and 781.5 eV that can be attributed to Co (III) and Co (II) [49]. 

**Figure 4:**
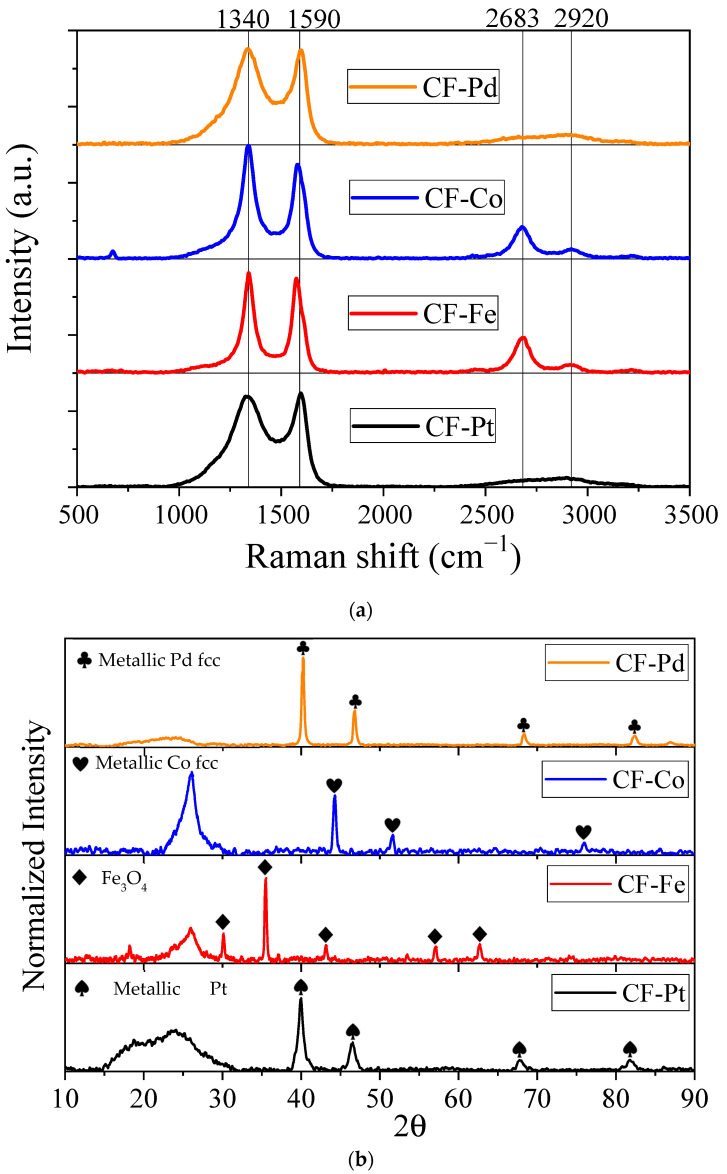
(**a**) Raman spectra of the carbon fibers of different metallic precursors. (**b**) X-ray diffraction patterns for the carbon fiber samples.

Figure 4a shows the Raman spectra of the lignin carbon fibers with different metal precursors, revealing the characteristic broad and overlapping bands at around 1340 and 1590 cm^−1^ corresponding to the so-called D and G bands of carbon materials, respectively. At the carbonization temperature used in this work, the ratio between the intensities of the two bands (I_D_/I_G_) does not apply as a measure of structural order, and other parameters, such as the narrowing of the D-band, should be considered [50]. The width of the D-band decreased in the order of CF-Pt > CF-Pd >> CF-Co > CF-Fe, depicting a notable increase in ordering for the transition-metal-loaded fibers. Compared to other works on lignin carbon fibers at the same carbonization temperature, the synthesized carbon fibers in this work show higher structural order (i.e., lower I_D_/I_G_ ratios and narrower D bands) [31,51]. It is also worth noting that the D-band in the palladium and platinum fibers had a higher amplitude towards wavenumbers near 1100 cm^−1^; this higher amplitude is attributed to the D4 band, related to carbon atoms with sp^3^ hybridization with low structural order [52]. The rearrangement of the carbon layers during the process may be hampered by the presence of uniformly distributed metallic particles in the fiber structure. The low structural order observed in these materials is associated with a high concentration of defects, which can be beneficial for the electrocatalytic activity of these fibers in the electrochemical reactions studied later [53].

Figure 4a also shows the Raman spectra between 2500 and 3000 cm^−1^, which contain information about the second-order modes. In this region, the band commonly referred to as 2D or G′~2700 cm^−1^ can be observed; this band is related to materials with electronic band structures like those in graphite-type materials [54]. A second peak known as the 2LO mode at ~2900 cm^−1^, corresponding to crystalline carbon structures [55], can also be observed. Interestingly, the materials have important differences in this region, especially in the 2D band, which has a much higher intensity for the Co- and Fe-containing carbon fibers. The high intensity of the 2D band for these materials indicates the presence of nanostructured carbon with an important local structural order, which is in agreement with the catalytic activity of these elements for graphitization processes [56,57]. Thus, from the Raman spectra analysis, it can be concluded that the carbon fibers obtained have a low degree of crystallinity. However, there were significant differences among the metals used in terms of the local structural order achieved by Fe and Co compared to Pd and Pt, with this parameter being higher for the Fe- and Co-containing materials.

Figure 4b shows the XRD patterns of the lignin-based carbon fibers with different metal precursors. For CF-Pd and CF-Pt, a broad peak centered at ca. 23° can be observed, associated with the presence of low-ordered carbon. However, the peak is narrower and shifted to ca. 26° in the Fe and especially in the Co-containing carbon fibers, resembling that of (002) of graphitic carbon (ICDD: 04-013-0293). A narrower (002) peak is indicative of a larger number of stacked graphene layers (Lc) in these samples. This finding confirms the Raman results concerning the presence of highly ordered carbon in the CF-Fe and CF-Co samples. In addition, further diffraction peaks corresponding to the presence of crystalline metal-containing phases were found. The platinum-containing carbon fibers presented diffraction peaks at 2θ of 39.9, 46.4, 67.7, and 81.6° corresponding to the fcc structure of metallic platinum (ICDD: 00-004-0802) [58]. In the case of the iron-containing carbon fibers, the XRD data reveal the pattern of an Fe_3_O_4_ structure with the five characteristic peaks at 30.1, 35.5, 43.1, 57.0, and 62.53° corresponding to the presence of a magnetite structure (ICDD: 01-071-6339) [59]. For the cobalt-containing carbon fiber, the XRD measurements confirm the existence of Co metal with an fcc structure owing to the finding of the three characteristic peaks at 44.3, 51.7, and 76.0° (ICDD: 15-0806) [60]. Finally, the diffraction peaks observed at 40.2, 47.5, 68.5, and 82.3° for the palladium-containing carbon fiber correspond to the fcc phase of metallic palladium (ICDD: 05-0681) [61]. To determine the metal-containing crystallite size using the Scherrer equation, the highest intensity peak for each sample at 2θ was used, with the result compiled in Table 3. It was found that the iron sample had the largest particle size, 35.8 nm, while platinum achieved the highest degree of dispersion (with a particle size of 10.6 nm).

**Figure 5:**
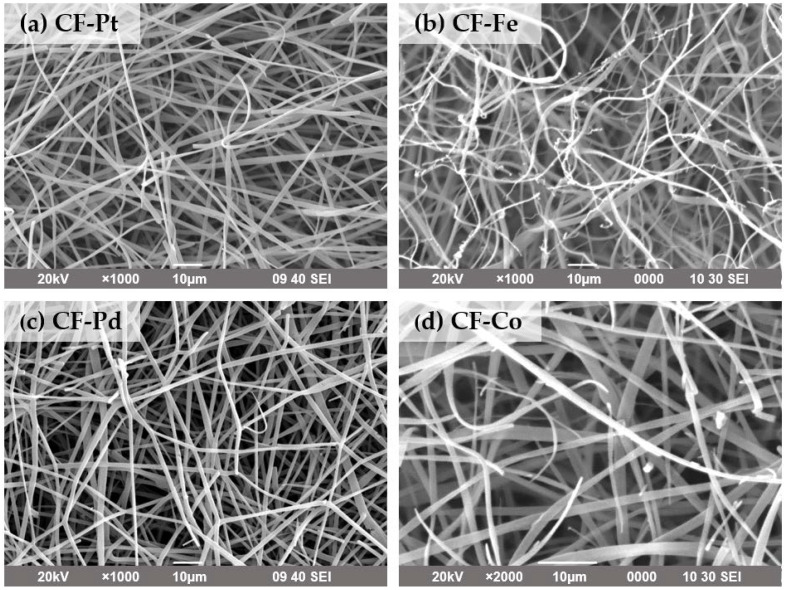
SEM images for the carbon fiber electrocatalysts: (**a**) CF-Pt; (**b**) CF-Fe; (**c**) CF-Pd; (**d**) CF-Co.

Figure 5 displays the SEM images of all the carbon fibers, where the formation of continuous fibers with diameter values from 1 to 3 μm was confirmed for all the samples. Average sizes of 1.60, 1.32, 1.58, and 1.62 μm were measured for CF-Pd, CF-Pt, CF-Fe, and CF-Co, respectively. The high aspect ratio of the carbon fibers ensured improved mass transfer, whereas the continuity of the fibers and the large number of contacts enhanced electrical conductivity [43]. Nevertheless, more defects as well as rougher surfaces were observed in the Fe-doped carbon fibers, and this finding is probably related to the formation of large nanoparticles (see average sizes measured via XRD in Table 3). 

**Figure 6:**
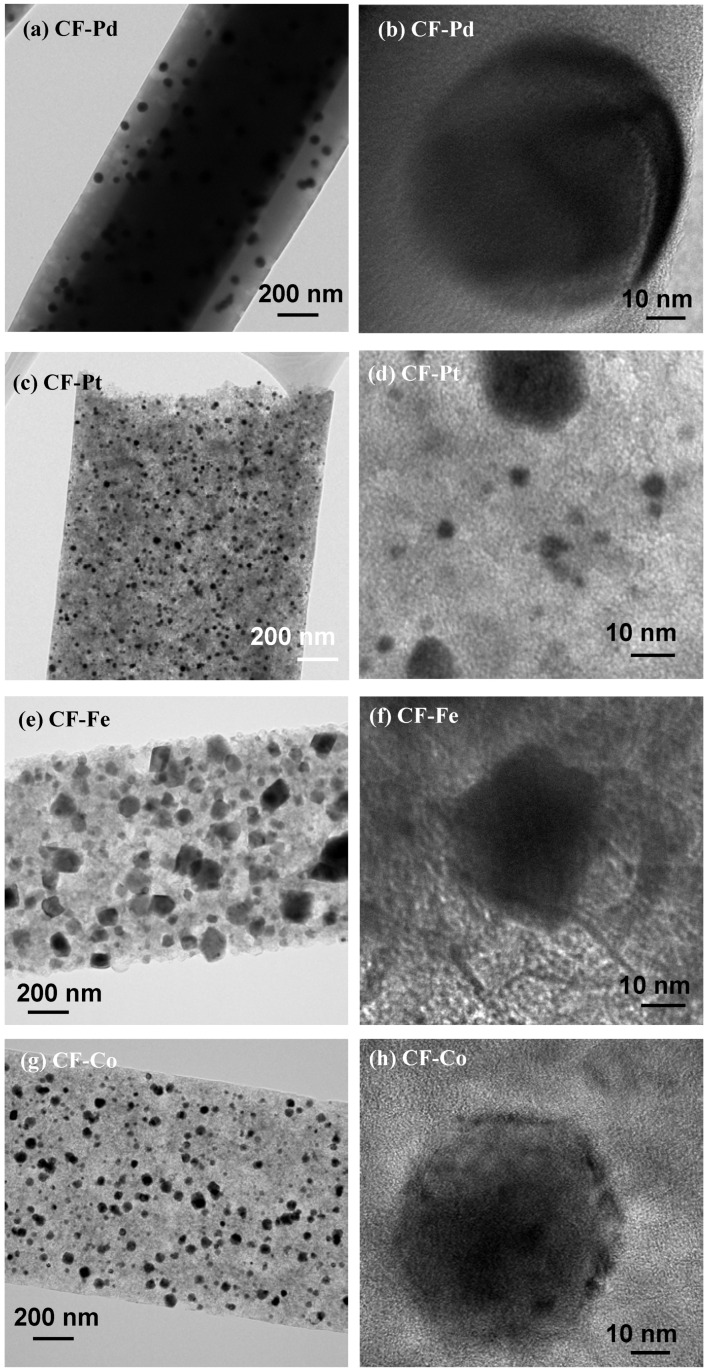
TEM images for the carbon fiber electrocatalysts: (**a**,**b**) CF-Pd; (**c**,**d**) CF-Pt; (**e**,**f**) CF-Fe; (**g**,**h**) CF-Co.

**Figure 7:**
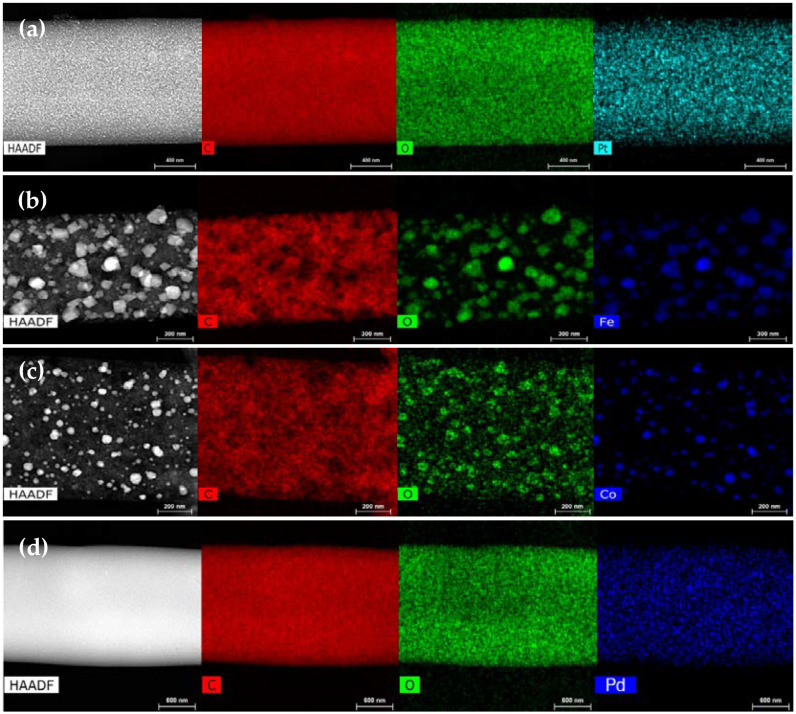
TEM mapping images for the carbon fiber electrocatalysts: (**a**) CF-Pt; (**b**) CF-Fe; (**c**) CF-Co; (**d**) CF-Pd.

The TEM images, presented in Figure 6 and Figure 7, indicate the existence of uniformly dispersed metal nanoparticles along the carbon fibers in all instances. However, the structure and morphology of the carbon fibers appear to be modified by the specific metal loaded in them. Thus, the CF-Pd samples show a smooth outer surface and nanosized regions of lower density (i.e., clearer holes) in the vicinity of the metal nanoparticles, suggesting the presence of mesopores formed by self-gasification reactions catalyzed by palladium. The high-resolution TEM image of CF-Pd, Figure 6b, reveals a disordered carbon structure around the crystalline metallic nanoparticles. The generation of mesopores seems to be higher for CF-Pt, as shown in Figure 6c,d, whereas a broader distribution of sizes for the Pt nanoparticles can be observed, with the larger nanoparticles revealing a defined geometry (Figure 6d). In contrast, the structures of the carbon matrices of the CF-Fe and CF-Co materials are much less dense, and the formation of ordered carbon nanostructures around the iron and cobalt nanoparticles, which was suggested by Raman and XRD analyses, is confirmed in the the HR-TEM image (Figure 6e–h).

The EDX mapping results in Figure 7 confirm that the metals particles’ distribution and average sizes are different, being more homogeneous and of smaller size in the case of noble metals (Figure 7a,d). Moreover, the agglomeration of cobalt and iron to form larger metallic particles was confirmed, with the nanoparticle size in CF-Fe showing a broader distribution than the rest of samples. In addition, for both cobalt and iron, the metal nanoparticles seem to be composed of metal oxides, as indicated by the similar patterns in the oxygen and Fe/Co mapping data in Figure 7b,c.

**Figure 8:**
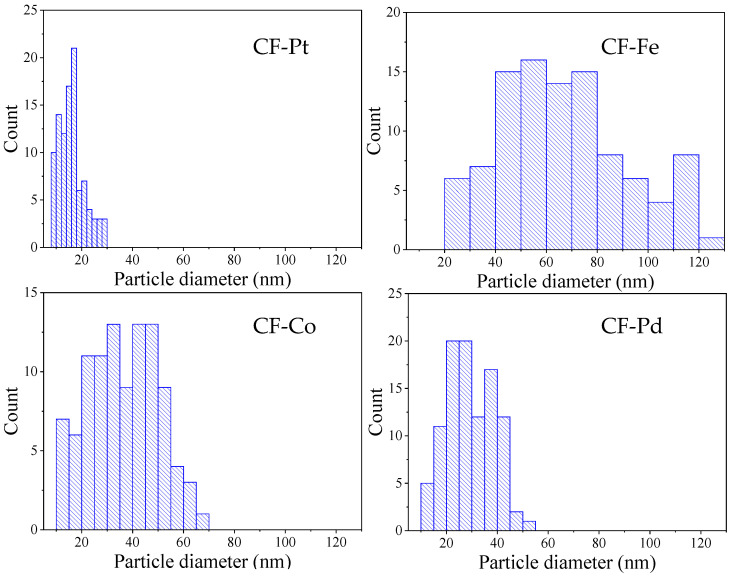
Histograms of particle size distributions of the metal-containing fibers.

Figure 8 shows the histograms of the particle size distribution of the metal-containing fibers. The sizes of the nanoparticles are between 8 and 30 nm for the Pt-containing fibers, 13 and 52 nm for the Pd-containing fibers, 13 and 66 nm for the Co-containing fibers, and 20 and 122 nm for the Fe-containing fibers. The average particle sizes are reported in Table 3. These results confirm the trends observed using XRD. 

### 3.3. Electrochemical Characterization

**Figure 9:**
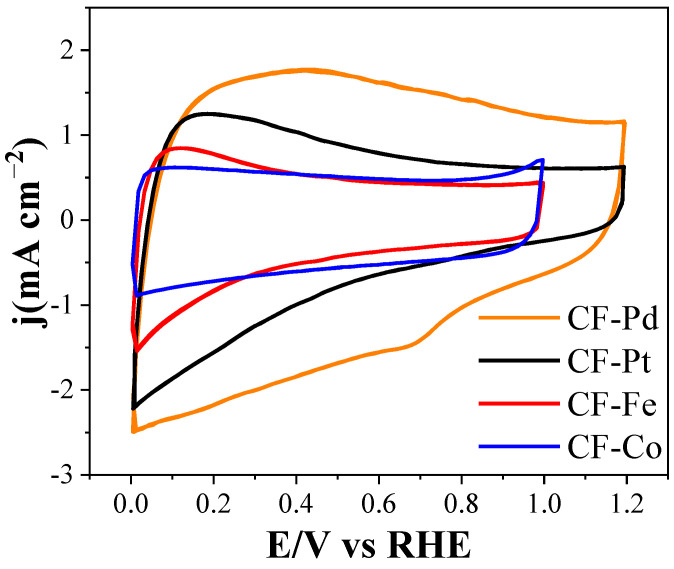
Steady state cyclic voltammograms during the 50th cycle for the carbon fibers in 0.1 M KOH, using a scan rate of 50 mV s^−1^, saturated with N_2_.

Figure 9 shows the electrochemical responses of the as-prepared lignin-based carbon fibers, which were tested using cyclic voltammetry in an alkaline electrolyte from 0 to 1 V for the cobalt- and iron-containing carbon fibers and between 0 and 1.2 V for the carbon fibers that contain noble metals in order to stabilize the responses and activate the metal surfaces [62]. All the samples exhibited a nearly rectangular shape, consistent with the formation of an electric double layer related to the capacitance of each material. This behavior aligns with the high electrical conductivity and well-developed surface area of the materials, both being desirable properties for improving the activity of the electrocatalysts. The materials with higher capacitance after the activation cycles are the palladium-containing carbon fibers, probably due to the predominant presence of micropores with sizes of ca. 0.7 nm (Figure 2b), which are known to be optimal for maximizing the capacitive response of carbon materials [63]. The strong capacitive response of porous carbon materials occludes the contribution of the Faradic processes associated with the presence of electroactive metals, such as those loaded on the surfaces of the studied electrocatalysts. Nevertheless, a cathodic current at a potential of ca. 0.7 V was observed during the negative sweep of the CF-Pd sample; it was attributed to the reduction of palladium oxide. After successively cycling in this range of potentials for, at least, 50 cycles, the low reactivity of the lignin-based carbon fibers was consistent with the electrochemical stability that all the samples exhibited. The electrochemical responses, determined via cyclic voltammetry, of the fiber dispersions are also presented in Figure S2. As can be seen, significantly higher current densities were achieved compared to the fibers attached directly to the electrode. This was due to improved interaction of the material with the collector.

**Figure 10:**
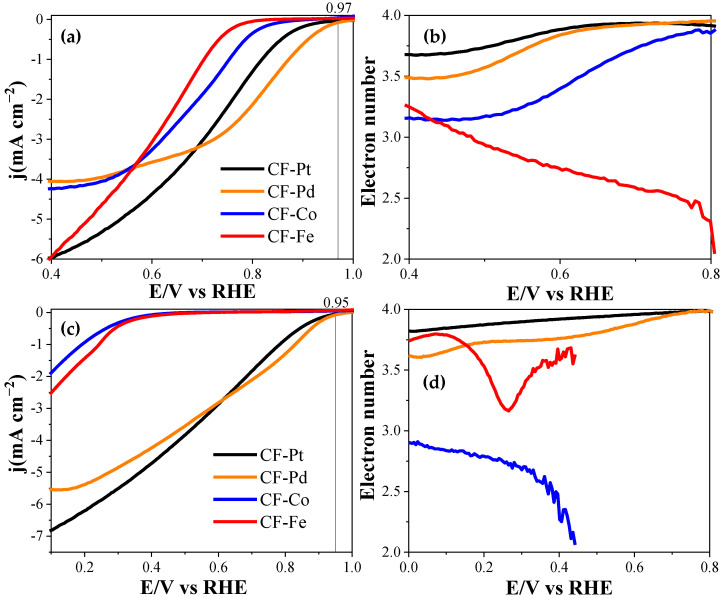
Linear sweep voltammograms obtained with the rotating disc electrode in (**a**) 0.1 M KOH and (**c**) 0.5 M H_2_SO_4_. Electron transfer number for the carbon fiber electrocatalysts in (**b**) 0.1 M KOH and (**d**) 0.5 M H_2_SO_4_. All tests saturated with O_2_ at 1600 rpm; scan rate of 5 mV s^−1^.

Figure 10 and Table 4 compile the electrochemical activity results with respect to the oxygen reduction reaction in acid and alkaline electrolytes for the different electrocatalysts prepared. It must be noted that the LSV measurements, taken at 1600 rpm, were directly performed using carbon fiber mats, resulting in poorer contact with the glassy carbon disk (the complete study of all scan rates is attached in Figure S1). For this reason, the LSV measurements exhibit a lower slope compared to measurements conducted in thin-film powders. In spite of this, the electrocatalytic activity of the materials is remarkable. The results indicate that the most active material for the oxygen reduction reaction was that obtained with the Pd precursor, followed closely by the CF-Pt sample. The former catalyst, studied in an alkaline medium, had a reaction onset potential of 0.97 V, surpassing the rest of the electrocatalysts, reaching a limiting current density of 4.1 mA cm^−2^. The number of electrons transferred, taken at a 0.7 V, was close to four in both acid and alkaline media, indicating its high selectivity for the four-electron pathway, the one most energetically efficient and preferred for energy storage and conversion devices. On the other hand, a significant improvement was observed in the behavior of the Fe- and Co-containing carbon fibers when studied in an alkaline medium, showing similar performance to that achieved by Ni- and Co-containing lignin-based carbon fibers, but without requiring the use of PEO to facilitate the electrospinning process [28]; however, they still present a much lower reaction onset potential compared to that obtained with the Pd-containing fiber electrocatalyst. CF-Fe and CF-Co also showed well-developed porosity, along with a high content of ordered nanostructured carbon material according to Raman and XRD measurements, which should enhance the conductivity of the electrodes. Therefore, the origin of the lower activity can be attributed to the larger particle size and the formation of pyrolytic carbon layers on top of the crystal surfaces, as seen in the TEM images and confirmed by the low metal content detected via XPS. The slightly superior performance of CF-Pd over CF-Pt could be related to the better metallic distribution (as depicted by the highest XPS/bulk metal loading (Table 4)) and the presence of wider mesopores in the former sample (Figure 2b), which might facilitate access of oxygen to the active phase. The ORR performance achieved using the fiber dispersions was also examined. The results are provided in Figure S3, where a similar trend to that obtained when using the fibers directly attached on the rotating ring disk electrode can be observed. In particular, the Pd-containing sample exhibits the highest onset potential, and it also follows the trend closest to the four-electron mechanism. 

Taking into account that the Pd-containing electrocatalyst used employs less than half the metal charge of the commercial material (8 vs. 20 wt%), this sample can be considered a promising replacement for the commercial Pt catalyst. The excellent performance was attributed to a synergistic effect between the lignin-based carbon fibers, whose micro-mesoporous structure allows for a very good distribution of the nanoparticles, resulting in excellent electrocatalytic activity, aided by the continuity of the carbon fibers, which allows for excellent electrical conductivity [31,43]. The lignin-based CF-Pd electrocatalyst outperforms similar lignin-derived electrospun carbon fiber electrocatalysts prepared using PEO or PVA as additives, such as those loaded with silver nanoparticles [29] or with manganese dioxide, and shows an onset potential similar to that of tri-doped B,N,F lignin-derived carbon fibers, which showed a comparable onset potential but a higher number of electrons transferred (3.85 vs. 4.00 (in this work)) [27], as they were only characterized for the ORR in an alkaline electrolyte. When compared with other Pt- and Pd-based electrospun electrocatalysts, the CF-Pd sample ranks in the middle-high tier when compared to supported catalysts (with onset potential values ranging from 0.89 up to 1.05 V vs. RHE) and reaches the third position in the self-supported catalyst ranking, with the high onset potential achieved in an acidic electrolyte being especially remarkable [20].

The value of *Cdl* was determined from half of the slope of the *Ja*–*Jc* vs. scan rate plot (Figure S4) and is summarized in Table S1, where *Ja* and *Jc* represent the anodic and cathodic currents, respectively, at a potential of 0.65 V vs. RHE in the case of CF-Fe, CF-Co and CF-Pt and at a potential of 0.85 V vs. RHE for the Pd-containing fibers. We observed that the Pd-containing and Pt-containing fibers presented a much higher double-layer capacitance, namely, 14.1 and 12.9 mF cm^−2^, respectively, than those obtained in the CF-Fe and CF-Co fibers, namely, 3.4 and 3.6 mF cm^−2^, respectively. These results are consistent with the porosity of the materials. 

**Figure 11:**
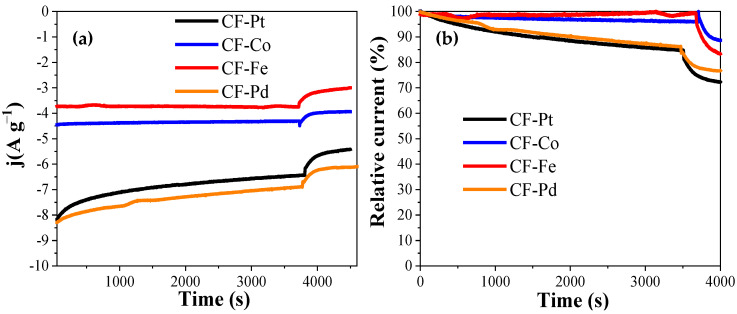
Comparative stability test for the different electrocatalysts at 0.65 V in O_2_-saturated 0.1 M KOH. Methanol was added after 60 min had elapsed. (**a**) Chronoamperometry. (**b**) Percentage of activity comparison.

The stability of the metal-containing carbon fibers was tested against the oxygen reduction reaction using a chronoamperometric technique (Figure 11). The test was carried using the same electrochemical setup used for the ORR activity determination in the O_2_-saturated 0.1 M KOH electrolyte, while the potential of the electrode was held at 0.65 V; i.e., the potential at which oxygen reduction was already initiated in all the samples. The data were normalized considering the mass of fiber used in the test. Figure 11a and Table 4 show that the CF-Pd sample had the highest activity during the whole period, showing a high level of stability similar to that of other self-supported catalysts [20].

After about 1 h, pure methanol was added to the electrolyte in order to reach a 1 M methanol concentration in the solution. It can be observed that in all the samples, there was a partial loss of activity, but none of them experienced complete poisoning by CO formed from the oxidation of methanol. Figure 11b shows the normalized remaining activity during the test, where it can be observed that the fibers based on noble metals suffered a more significant loss of activity than the iron and cobalt fibers. According to these results, carbon fibers made from lignin exhibit outstanding behavior against CO poisoning and good electrocatalytic performance.

**Figure 12:**
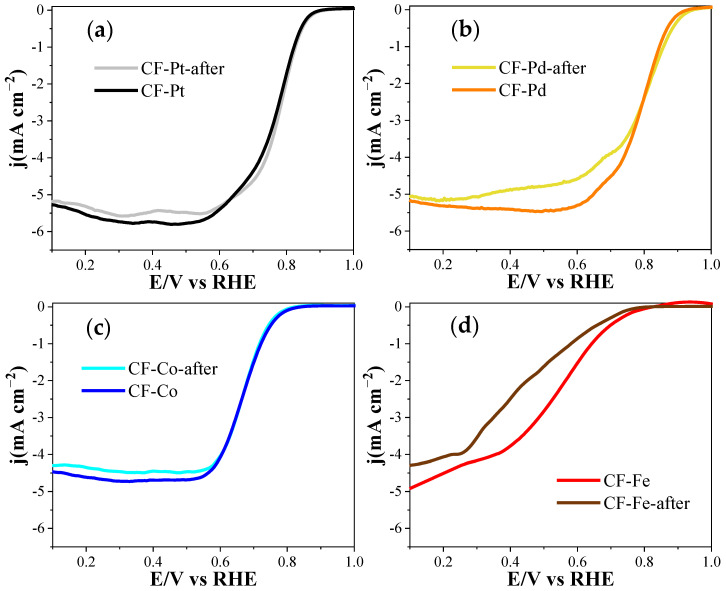
Linear sweep voltammograms for the metal-containing carbon fiber inks before and after the stability test with 500 cycles: (**a**) CF-Pt, (**b**) CF-Pd, (**c**) CF-Co, and (**d**) CF-Fe.

The stability of the materials in the ORR was also examined through a cycling stability test (500 cycles, see Figure 12). In this case, the test was performed with inks prepared from the metal-containing carbon fibers. In all samples, a slight decrease in activity was observed, with activity losses of 5.8%, 5.4%, 3.1%, and 2.7% for CF-Fe, CF-Co, CF-Pt, and CF-Pd, respectively, evaluated at 0.2 V. These results indicate that the samples maintained a good degree of stability in terms of the ORR. In addition, we present XPS data taken before and after the electrochemical measurements (Figure S5). As observed in these spectra, small changes can be distinguished, which mainly correspond to an increase in higher-oxidation-state metal species. These results are in agreement with the observed high stability of the materials. 

The results of the EIS for the fiber dispersions are shown in Figure S6. The metal-containing carbon fibers were compared with the carbon fiber without any metal species incorporation. Interestingly, a significant decrease in resistance was observed when metals were added. The impedance spectra for each metal-containing carbon fiber sample before and after the electrochemical cycling stability test are shown in Figure S7. It can be concluded that there are no important changes in the spectra, thus supporting the excellent electrochemical stability of the materials studied.

Although the use of lignin-based carbon fibers as ORR electrocatalysts has been proposed in the literature [64], to the best of our knowledge, only four studies have explored such an application. In the seminal work by Lai et al., Ag supported on lignin-derived electrospun carbon fibers was tested as an ORR catalyst using an RDE in a 0.1 M KOH solution, revealing an onset potential of 0.78 V vs. RHE [29]. More recently, Co and Ni supported on carbon fiber catalysts were prepared using a one-pot procedure, reaching onset potentials of ca. 0.82 V vs. RHE in 0.1 M of KOH and halfwave potentials of 0.79 V vs. RHE [28]; however, PEO was added as a binder. Similarly, Mn-based ORR electrocatalysts were prepared by electrospinning lignosulfonate, PVA, and KMnO4 mixtures, resulting in lower ORR activities than the samples reported herein [30]. Finally, ORR onset potentials similar to those reported for the catalysts analyzed herein have been published, although the use of PVP as a binder and additives such as zinc borate or ammonium fluoride was needed [27].

## 4. Conclusions

Without the use of any structural stabilizing agents, freestanding porous carbon fibers containing different supported metal nanoparticles were efficiently produced in just one stage through the electrospinning of lignin solutions. The weight of the incorporated metal-containing nanoparticles varied from 8 to 16 wt%, and the nanoparticles exhibited a very good distribution throughout the fibers. Moreover, adding metal precursors to the lignin solution also promoted the induction of mesoporosity in the resulting metal-containing carbon fibers. This is an attractive characteristic in terms of increasing the accessibility of the active sites without reducing the electrical conductivity when the fibers are used as electrocatalysts. Our electrochemical analysis demonstrated that these metal-containing carbon fibers are potential ORR catalysts. The Pd-containing carbon fibers stand out for their exceptional electrochemical activity and preference for the four-electron route in both acidic and alkaline conditions. Based on these results, lignin-based carbon fibers obtained via electrospinning with the incorporation of metal nanoparticles without using additives or binders demonstrate promising electrocatalytic activity, making them a viable substitute for current commercial Pt/C ORR catalysts in acidic and alkaline media. These materials could be directly used as freestanding electrodes in fuel cells or metal air batteries.

## Figures and Tables

**Table 1:** Preparation yields of the samples.

Sample	Stabilization Yield (wt%)	Carbonization Yield (wt%)	Overall Yield (wt%)
CF-Pt	45	22	9.8
CF-Co	53	16	8.5
CF-Fe	33	25	8.3
CF-Pd	56	30	16.8

**Table 2:** Porosity and chemical composition of samples.

	N_2_ Adsorption Isotherm			XPS (wt%)						Bulk Metal Loading (XRF) wt%
Sample	A_BET_ m^2^ g^−1^	V_t_ cm^3^ g^−1^	V_mes_ cm^3^ g^−1^	C	O	Pt	Fe	Co	Pd	
CF-Pt	785 ± 2	0.18 ± 0.04	0.51 ± 0.06	90.4	2.5	7.1	--	--	--	10
CF-Co	500 ± 1	0.04 ± 0.01	1.10 ± 0.10	93.2	3.2	--	--	3.4	--	16
CF-Fe	350 ± 1	0.04 ± 0.01	0.59 ± 0.05	91.5	3.8	--	4.3	--	--	16
CF-Pd	755 ± 2	0.23 ± 0.03	0.39 ± 0.04	90.2	2.6	--	--	--	7.2	8

**Table 3:** Crystallite size for the carbon fiber samples obtained via XRD and average nanoparticle size obtained via TEM.

Sample	2θ	Crystallite Size (Scherrer eq, nm)	Average Nanoparticle Size Determined via TEM (nm)	Nanoparticle Size Range Determined via TEM (nm)
CF-Pd	40.22	25.2	29.4	13.2–52.4
CF-Co	44.26	24.0	36.2	12.7–65.8
CF-Fe	35.48	35.8	67.3	20.1–122.1
CF-Pt	39.93	10.6	16.1	8.2–29.6

**Table 4:** ORR electrochemical parameters in alkaline and acidic electrolytes. Current obtained in the stability test in alkaline medium.

Alkaline Medium			
Electrocatalyst	E (Onset) (V) (at j = 0.1 mA cm^−2^)	*n* (at 0.7 V)	Current After 30 min (A g^−1^)
CF-Pd	0.97	4.0	7.3
CF-Co	0.85	3.8	4.4
CF-Fe	0.79	2.6	3.7
CF-Pt	0.93	3.9	6.8
**Acidic Medium**			
**Electrocatalyst**	**E (Onset) (V) (at j = 0.1 mA cm^−2^)**	***n***	
CF-Pd	0.95	4.0 (at 0.7 V)	
CF-Co	0.42	2.7 (at 0.3 V)	
CF-Fe	0.46	3.3 (at 0.3 V)	
CF-Pt	0.95	4.0 (at 0.7 V)	

## Data Availability

Data are contained within the article.

## Supplementary Materials

The following supporting information can be downloaded at https://www.mdpi.com/article/10.3390/nano13222921/s1. Figure S1. LSVs at different rotation speeds for: (a) CF-Co, (b) CF-Fe, (c) CF-Pt and (d) CF-Pd. Figure S2. Steady state cyclic voltammograms during the 20th cycle for the metal-containing carbon fibers inks in 0.1 M KOH, scan rate of 50 mV s^−1^, saturated with N_2_. Figure S3. ORR performance for the metal-containing carbon fibers inks in O2-saturated 0.1 M KOH at 1600 rpm. Scan rate of 5 mV s^−1^. (a) Linear sweep voltammograms. (b) Electron transfer number. Figure S4. Ja–Jc vs. scan rate plot for the different electrocatalysts. Figure S5. XPS spectra of the metal-containing carbon fibers dispersions before and after the electrochemical stability test of the 500 cycles. (a) CF-Pt, (b) CF-Pd, (c) CF-Co and (d) CF-Fe. Figure S6. Impedance spectra for the metal-containing carbon fibers inks using a N_2_-saturated 0.1 M KOH, at 0.2 V vs RHE, frequencies from 6000 to 0.1 Hz. Figure S7. Impedance spectra for each metal-containing carbon fiber ink before and after the cycling voltammetry. (a) CF-Pt, (b) CF-Pd, (c) CF-Co and (d) CF-Fe. Table S1. Doble layer capacitance value for each carbon fiber inks and TOF.
