# Supplementary material for: Sustainable Synthesis of Metal-Doped Lignin-Derived Electrospun Carbon Fibers for the Development of ORR Electrocatalysts
Source: Nanomaterials (Basel). 2023 Nov 9;13(22):2921. doi: 10.3390/nano13222921 (PMC10674835; doi:10.3390/nano13222921)
Supplement: Supplementary file 1 [file nanomaterials-13-02921-s001.zip › nanomaterials-2668728-supplementary.pdf]

Supporting information

Sustainable Synthesis of Metal-Doped Lignin-derived Electrospun Carbon Fibers for the Development of ORR Electrocatalysts

Cristian Daniel Jaimes-Paez [1], Francisco José García-Mateos [2], Ramiro Ruiz-Rosas [2,*] , José Rodríguez-Mirasol [2] , Tomás Cordero [2] , Emilia Morallón [1] and Diego Cazorla-Amorós [3,*]

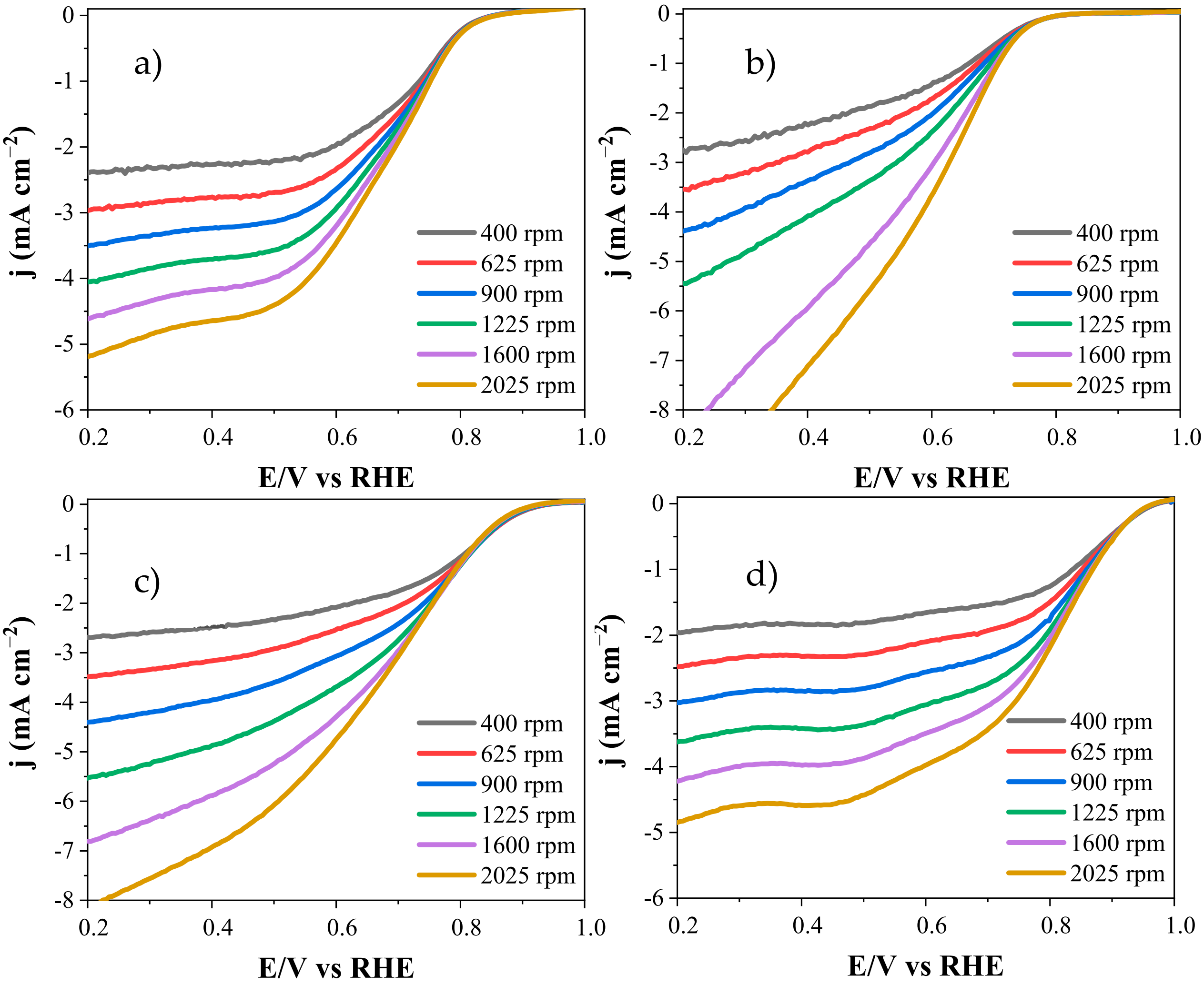

Figure S1. LSVs at different rotation speeds for: a) CF-Co, b) CF-Fe, c) CF-Pt and d) CF-Pd.

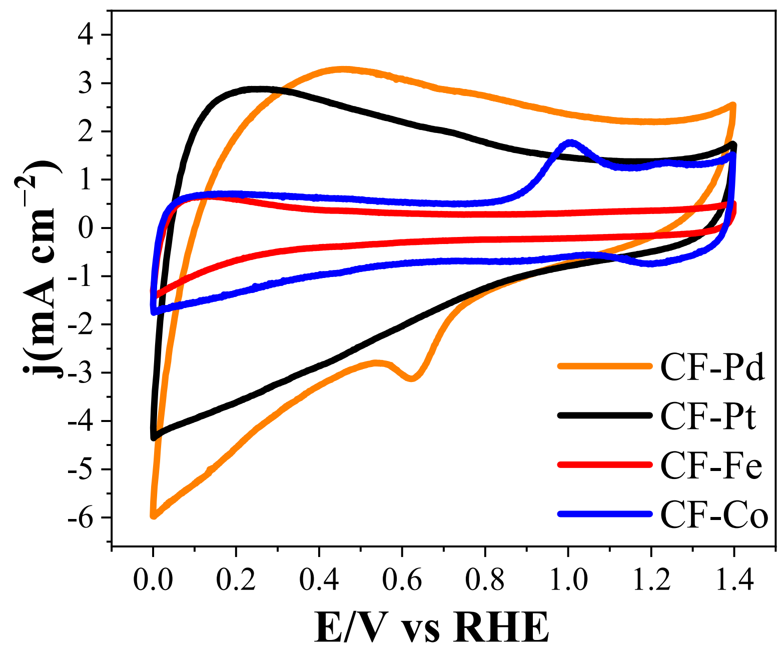

Figure S2. Steady state cyclic voltammograms during the 20th cycle for the metal-containing carbon fibers inks in 0.1 M KOH, scan rate of 50 mV s^{-1}, saturated with N_2.

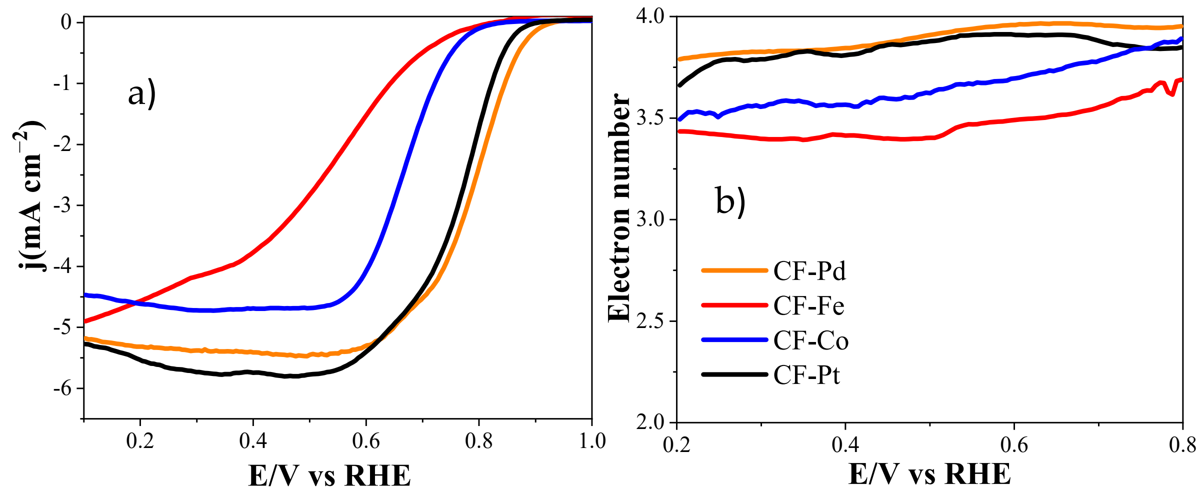

Figure S3. ORR performance for the metal-containing carbon fibers inks in O_2-saturated 0.1 M KOH at 1600 rpm. Scan rate of 5 mV s^{-1}. a) Linear sweep voltammograms. b) Electron transfer number.

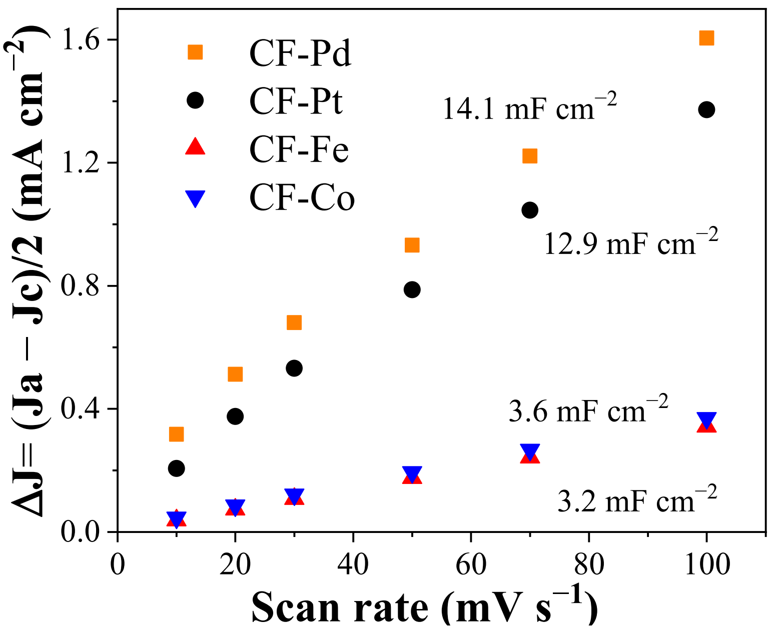

Figure S4. Ja–Jc vs. scan rate plot for the different electrocatalysts.

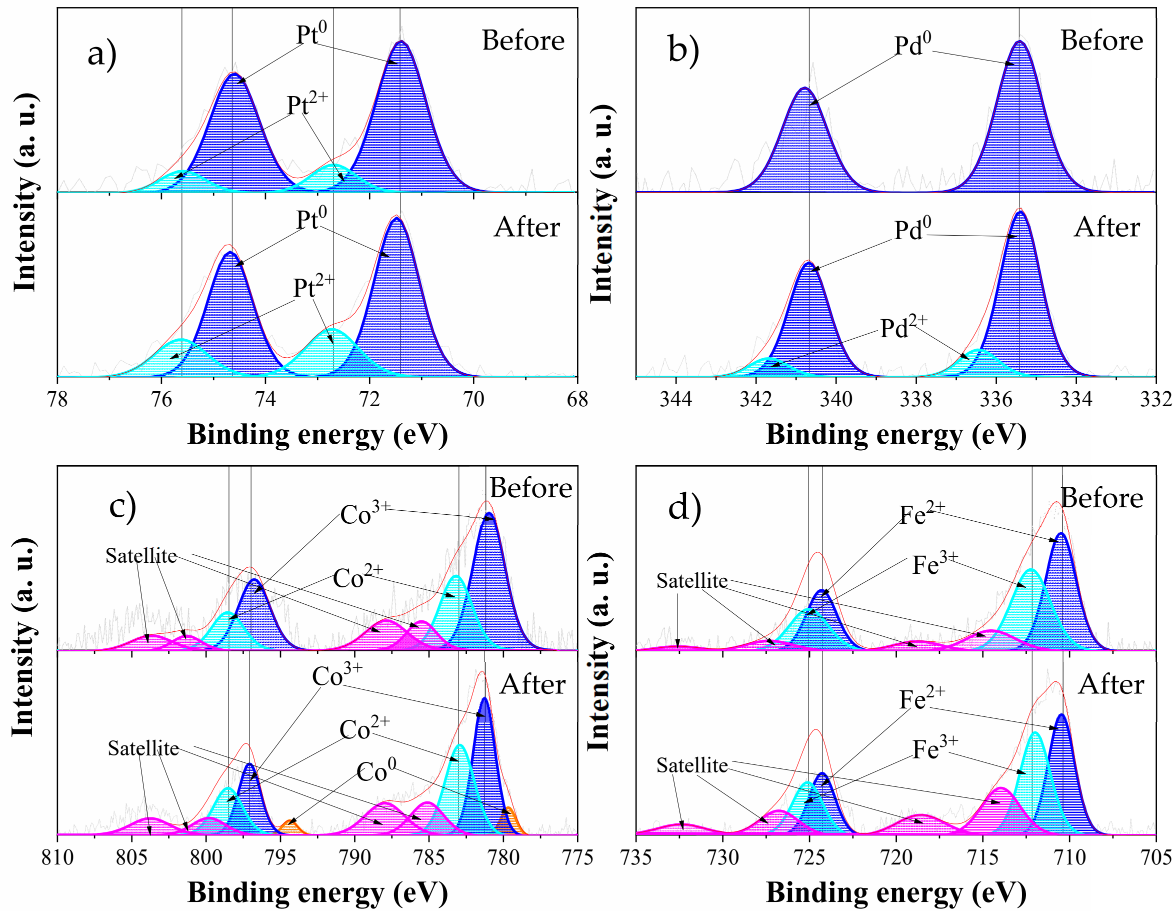

Figure S5. XPS spectra of the metal-containing carbon fibers dispersions before and after the electrochemical stability test of the 500 cycles. a) CF-Pt, b) CF-Pd, c) CF-Co and d) CF-Fe.

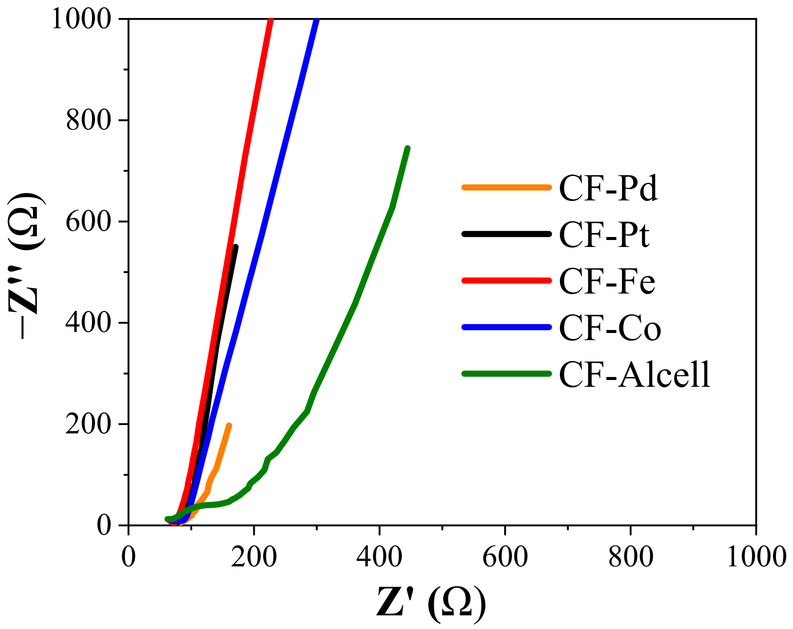

Figure S6. Impedance spectra for the metal-containing carbon fibers inks using a N_2-saturated 0.1 M KOH, at 0.2 V vs RHE, frequencies from 6000 to 0.1 Hz.

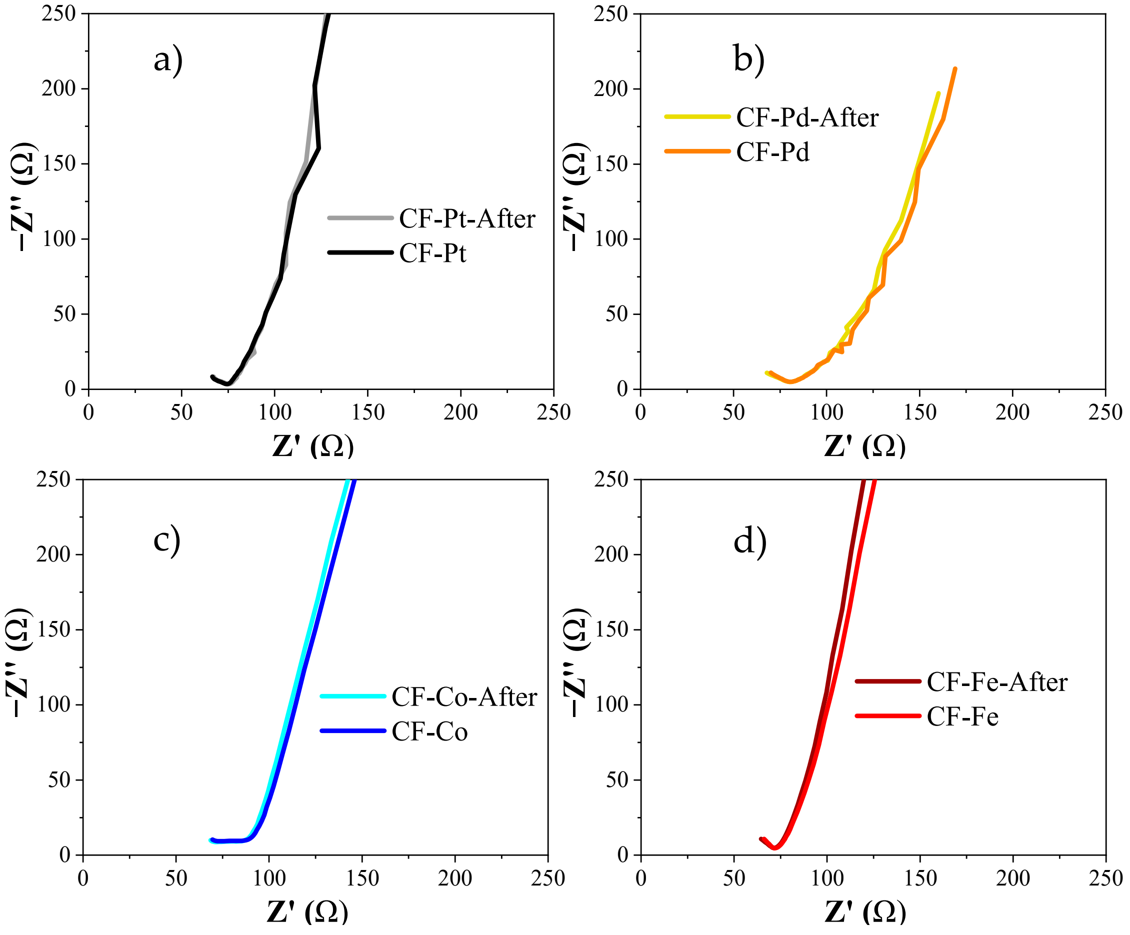

Figure S7. Impedance spectra for each metal-containing carbon fiber ink before and after the cycling voltammetry. a) CF-Pt, b) CF-Pd, c) CF-Co and d) CF-Fe.

Table S1. Doble layer capacitance value for each carbon fiber inks and TOF.

Sample	*Cdl* (at 0.65 V)	TOF (s^{-1})*
**CF-Pd**	14.1 (at 0.85 V)	0.037
**CF-Co**	3.6	0.029
**CF-Fe**	3.4	0.034
**CF-Pt**	12.9	0.073

* TOF has been calculated following the equation:

TOF = (I / *n*F) / M

where:

- TOF is Turnover Frequency (s^{-1}).
- I experimental current at 0.4 V.
- *n* is the electron transfer number involved in the ORR at 0.4 obtained from Figure S3.
- F is the Faraday constant
- M is the mol of each metal in the carbon electrode as determined from XPS
